# The role of high-intensity physical exercise in the prevention of disability among community-dwelling older people

**DOI:** 10.1186/s12877-016-0334-y

**Published:** 2016-11-09

**Authors:** Astrid Etman, Frank H. Pierik, Carlijn B. M. Kamphuis, Alex Burdorf, Frank J. van Lenthe

**Affiliations:** 1 Department of Public Health, Erasmus University Medical Centre, Rotterdam, The Netherlands; 2 Department of Urban Environment and Safety, TNO, Utrecht, The Netherlands; 3 Department of Human Geography and Spatial Planning, Utrecht University, Utrecht, The Netherlands

**Keywords:** Disability, Physical activity, Sports, Elderly

## Abstract

**Background:**

Moderate to vigorous physical activity (MVPA) is considered important to prevent disability among community-dwelling older people. To develop MVPA programs aimed at reducing or preventing disability more insight is needed in the contributions of exercise duration and intensity and the interplay between the two.

**Methods:**

Longitudinal data of 276 Dutch community-dwelling persons aged 65 years and older participating in the Elderly And their Neighbourhood (ELANE) study were used. MVPA exercise (yes/no), duration (hours per two weeks), intensity (Metabolic Equivalent of Task; METs), and energy expenditure (MET-hours per two weeks), and disability in instrumental activities of daily living (range 0–8) were measured twice within 9 months to account for fluctuations over time. Associations between the four exercise measures and disability were tested with longitudinal tobit regression analyses.

**Results:**

MVPA exercise was associated with fewer disabilities. While exercise duration was not associated with disability, whereas an increase of one MET in exercise intensity was associated with 0.14 fewer disabilities (95 % CI: -0.26 to -0.02). For exercise energy expenditure, an increase of one MET-hour exercise per two weeks was associated with 0.03 fewer disabilities (95 % CI: -0.05 to -0.01).

**Conclusions:**

Higher-intensity exercise may help to prevent disability among community-dwelling older people. Further investigation is needed to explore the preventive effects in more detail.

## Background

Preventing disability is of major importance in ageing societies. From 17 to 54 % of community-dwelling people aged 65 years and older suffer from one or more disabilities in daily activities [1–3], which may result in a loss of independent living and increased healthcare costs. At older age, maintaining an active lifestyle through regular moderate to vigorous physical activity (MVPA) may delay age-related decline in physical functioning [4, 5]. MVPA induces physiological cardiovascular adaptations (e.g. better vessel wall function and structure), improves physical performance through better balance and muscle strength, and as such may prevent loss of function [6–8]. Informing health-related policy and practice on key elements of interventions to stimulate MVPA among older people is essential to accomplish the largest health gains. MVPA programs are increasingly offered to older people [9, 10]. However, the optimal “volume” (frequency, duration and intensity of exercise) to prevent disabilities is still unclear [11]. MVPA at increased duration, greater frequency, and/or higher intensity has been found most beneficial for many health outcomes [12]. However, little is known about the independent contributions of physical exercise duration and intensity, and their interplay in the prevention of disabilities [13, 14], which was investigated in this study.

## Methods

### Subjects

Longitudinal data from the Dutch Elderly And their Neighbourhood (ELANE) study (2011–2013) were used. The ELANE study aimed at studying associations between area characteristics and physical activity, independent living, and quality of life among community-dwelling people aged 65 years and older living in Spijkenisse, a middle-sized town in the greater Rotterdam area. The exclusion criteria were: institutionalised, bedridden, wheelchair- or scooter-bound, or not fluent in Dutch. Of the 430 participants interviewed face-to-face at the first time-point (T0; September 2011 – July 2012), 277 agreed to a second interview by telephone nine months later (T1; June 2012 – April 2013). Winter months were excluded from data collection. Only data of participants interviewed both at T0 and T1 were used. Because T1 data on disabilities were lacking for one person, data of 276 persons were eligible for analysis. Details of the ELANE study are provided elsewhere [15].

### Disabilities

Presence of disabilities was measured with the reliable Lawton and Brody functional ability scale [16, 17]. Disabilities among older people can be episodic and recurrent [17], which can be captured by repeated measurements. Participants were asked at both T0 and T1 whether they needed help with the following eight Instrumental Activities of Daily Living (IADL): using the telephone, travelling (e.g. public transport), grocery shopping, preparing a meal, household tasks, taking medicines, finances, and doing laundry. All items had the response categories ‘no’ (0) and ‘yes’ (1) and therefore the total score could range from 0 to 8.

### MVPA exercise

Both at T0 and T1, questions from the Physical Activity Questionnaire of the LASA-study (LAPAQ), a valid and reliable instrument specifically developed for older people [18, 19], served to determine four exercise measures. MVPA *exercise participation* was based on the question ‘Do you physically exercise?’ with response categories ‘yes’ (1) or ‘no’ (0). If the answer was ‘yes’, the following questions was asked related to a maximum of two exercise activities on which they spent most time: ‘In which type of physical exercise did you participate in the previous two weeks?’, ‘How often did you do this exercise in the previous two weeks?’, and ‘For how long did you usually do this exercise in the previous two weeks (minutes)?’. *Exercise duration* (hours) was calculated by multiplying the frequency with the total amount of time participating in exercise divided by 60. *Exercise intensity* was measured with the Metabolic Equivalent of Task (MET) (highest MET if two types of exercise were reported with different METs) based on the Compendium of Physical Activities in which exercise-specific intensities are listed as multiplies of the resting metabolic rate of 1.0 kcal/kg/h [20]. *Exercise energy expenditure* (MET-hours) was calculated by multiplying exercise duration by intensity. Exercise duration and exercise energy expenditure were each summed for the maximum of two types of exercise. As MVPA exercises by definition are exercises with an intensity of three or more METs [21], participants reporting exercises with intensities lower than 3 METs were categorized as not participating in MVPA exercises.

### Statistical analyses

Differences in sex, age, disabilities, and exercise participation between the study sample and persons lost to follow-up were tested with Chi-square tests and t-tests. The association between exercise intensity and duration was tested with a Pearson correlation.

Of the study sample, 72.8 % reported to have no disabilities at T0 and/or T1. Although this suggests that many persons did not experience any limitations there still may be subtle differences in IADL-performance among these persons. We therefore applied tobit regression analyses, an elegant way of analysing such censored data [22]. The *longitudinal* tobit method was used to handle data from two time-points (see the Appendix). Associations of the four exercise measures with disabilities were tested (sex- and age-adjusted) using STATA 13.1. A linear association between exercise duration and disabilities was found; therefore those who did not participate in exercise remained in the analyses. Educational level was not associated with disability. Additionally, adjustment for educational level did not change the results essentially, and educational level was therefore excluded from the analyses.

## Results

### Descriptive findings

Age, number of disabilities, and exercise participation did not differ between those who participated at both time points (study sample) and those who only participated at T0. The latter sample had a higher proportion of women.

In the study sample, at both T0 and T1, about one third reported to have one or more disabilities (Table 1). More disabilities were found among women and with increasing age (*p* < 0.05). While the number of disabilities had not changed between T0 and T1, exercise duration, intensity, and energy expenditure had all decreased. The proportion of persons participating in MVPA exercise was 46.4 % at T0 (*n* = 128) and 40.2 % at T1 (*n* = 111). Fitness, gymnastics (e.g. balance training), cycling on a stationary bike, and cycling tours were the most prevalent exercise types, and most respondents reported one type only (Table 2). Exercise duration and exercise intensity were positively correlated (*r* = 0.60; *p* < 0.001).

**Table 1:** Characteristics of the study sample aged 65 years and older participating in the ELANE study (*N* = 276)

			T0	T1
	Sex	(% women)	48.2	
	Mean age	(years)	74.6 ± 6.7	
	Disabilities (range 0–8)	(% one or more)	33.3	36.6
		(mean)	0.7 ± 1.4	0.8 ± 1.3
MVPA exercise^a^	Participation	(% yes)	46.4	42.4
	Mean duration	(hours in 2 weeks)	2.4 ± 4.6	2.1 ± 4.9**
	Mean intensity ^b^	(METs)	2.7 ± 3.1	2.3 ± 2.9**
	Mean energy expenditure	(MET-hours in 2 weeks)	14.1 ± 32.8	11.7 ± 30.1**

**p < 0.001

^a^all MVPA means are among the total study population

^b^mean score of T0 and T1 (in case participants participated in two different types of exercise, highest METs of both was used)

**Table 2:** Nature of MVPA exercise at T0 and T1 among older people participating in the ELANE study

		T0		T1	
	Intensity (METs)	Exercise 1 (*n* = 128)	Exercise 2 (*n* = 37)	Exercise 1 (*n* = 111)	Exercise 2 (*n* = 21)
Fitness	5.5	17.2 %	13.5 %	23.4 %	33.3 %
Gymnastics	4.0	13.3 %	8.1 %	8.1 %	19.0 %
Cycling on stationary bike	5.5	10.9 %	8.1 %	9.9 %	0.0 %
Cycling tours	8.0	8.6 %	24.3 %	3.6 %	19.0 %
Swimming	7.0	8.6 %	13.5 %	10.8 %	4.8 %
Dancing	4.5	6.3 %	2.7 %	6.3 %	9.5 %
Other	3.0–10.0	35.1 %	29.8 %	37.9 %	14.4 %

### Physical exercise and disability

Those participating in MVPA exercise reported 0.96 fewer disabilities than those not participating in MVPA exercise (Table 3, model 1). An increase in exercise duration and an increase in intensity were both associated with a decrease in disabilities (models 2 and 3). The association between exercise duration and disabilities became non-significant after adjustment for exercise intensity (model 4). Independent of exercise duration, a one MET higher intensity was associated with 0.14 fewer disabilities (model 5). A one MET-hour increase was associated with 0.03 fewer disabilities (model 6).

**Table 3:** Age- and sex-adjusted associations between MVPA exercise measures and disabilities among community-dwelling older people, ELANE study (*N* = 276)

Model	β	(95 % CI)	*p*-value
1. Exercise participation (yes/no)	−0.96	(-1.53 to -0.39)	0.001
2. Exercise duration (hours per 2 weeks)	−0.09	(-0.17 to -0.01)	0.034
3. Exercise intensity (METs)	−0.16	(-0.27 to -0.06)	0.002
4. Exercise duration (hours per 2 weeks), adjusted for intensity	−0.03	(-0.12 to 0.06)	0.508
5. Exercise intensity (METs), adjusted for duration	−0.14	(-0.26 to -0.02)	0.021
6. Exercise energy expenditure (MET-hours per 2 weeks)	−0.03	(-0.05 to -0.01)	0.002

## Discussion

Participation in MVPA exercise was associated with fewer disabilities. Exercise intensity had a stronger, negative association with disabilities than had exercise duration. When both exercise duration and intensity were taken into account, no association was found for duration, whereas higher intensity was associated with fewer disabilities. Exercise energy expenditure was also associated with fewer disabilities.

### Strengths and limitations

This study is among the first to investigate the role of MVPA exercise duration, intensity, and the interplay between both in relation to disability, which information is highly relevant for exercise programs aimed at reducing or preventing disabilities among community-dwelling older people. A key strength is the use of repeated measures, which provides more robust associations than the use of a single measure in cross-sectional designs. Although differences in other health-related factors cannot be ruled out, the factors age, number of disabilities, and exercise participation did not differ between the study population and those only participating at T0. We think, therefore, that there is only a small probability that a ‘survival group’ was interviewed.

A limitation of this study is that disabilities and exercise participation levels were self-reported, with the inherent risk of measurement error [23, 24]. However, particularly for organized exercise activities conducted at predetermined hours and days per week (as reported by a substantial proportion of the study sample), reporting may be relatively easy and therefore less prone to bias. A methodological limitation is that participants were asked to report on a maximum of two exercise activities. To what extent this has led to an underreporting of MVPA is unclear, considering we do not know how many people actually participated in more than two exercise activities. Furthermore, we cannot rule out seasonal influences, although the reported decrease in exercise duration and increase in indoor sport activities in the summer makes it unlikely that our findings are affected by the difference in seasons at T0 and T1. Another limitation is that an exercise can be performed at different levels of intensity and consequently with different energy expenditure [25], which has not been taken into account. Measuring exercise intensity objectively, for example by using heart rate monitors or accelerometers [26, 27], would introduce further precision about the intensity of exercise.

### Discussion of findings

Older people participating in MVPA exercise reported fewer disabilities than those not participating in MVPA exercise, which can be explained by two mechanisms: 1) persons experiencing disabilities are less likely to engage in MVPA exercise [28]; and/or 2) participating in MVPA exercise may prevent older people from developing disabilities [29, 30]. While the use of repeated measures allowed minimizing the impact of the episodic nature of disabilities, testing the direction of the association may require a longer study period (including multiple measurements) in which persons start to engage in exercise and develop disabilities.

The association between exercise duration and disabilities may be overestimated when intensity is not taken into account. This is in line with the finding that higher exercise intensity was associated with fewer disabilities, and that persons participating in higher-intensity exercise tended to exercise longer than did persons participating in lower-intensity exercise. Energy expenditure was weakly associated with disabilities. This can be largely attributed to exercise intensity, also considering that a systematic review found that high-intensity exercise programs have a positive effect on disabilities [13]. This indicates that besides evidence of an inverse association between physical activity and disability, intervening on disability by offering MVPA programs seems promising [13, 31].

### Implications

The results suggest that higher-intensity exercise (e.g. swimming or fitness) may be more effective in preventing functional loss among older people than lower-intensity exercise (e.g. gymnastics or dancing). The finding that one MET-hour higher exercise energy expenditure was associated with 0.03 few disabilities may implicate that for example an increase of 3 MET-hours per two weeks, which can be realized by 35 min fitness exercise (at 5.5 METs; per two weeks), may decrease disabilities with 0.1. Arguably speculative, this would have a positive effect on independent living as one would have less difficulty with activities of daily life. As 17 to 54 % of the over 65 year olds suffer from one or more disabilities and disability-associated health care expenditures accounts for 26.7 % of all health care expenditures [32], the effect may be rather substantial. It would be of interest to investigate what activities of daily living would benefit most of higher-intensity exercise, and how this would affect health care costs.

Other studies support clear fitness, metabolic, and performance benefits of higher-intensity MVPA, although the MVPA programs not necessarily need to be of highest intensities to reduce health risks [12]. Exercise participation recommendations for persons already experiencing disabilities should be made with caution, since high-intensity exercise participation for this group may not be feasible [21].

## Conclusion

Higher-intensity exercise may help to prevent disability among community-dwelling older people. Further investigation is needed to explore the preventive effects in more detail.

## Appendix

Application of longitudinal tobit regression analyses. This appendix describes the application of longitudinal tobit regression analyses that was used to handle the data and the reasoning behind choosing this method.

**Background**

The main reasons why we chose to use tobit regression analyses were:

- Censored data
  A large part of participants reported to have no disabilities, which suggests that many persons did not experience any limitations. However, there still may be subtle differences in IADL-performance among these persons. The use of tobit regression analyses allows to analyse censored data.
- Fluctuations in disability level
  Since disability level can fluctuate over time, using data from two time-points is preferred over generally used cross sectional designs. Therefore, the *longitudinal* tobit method was used to handle disability data from two time-points.

**Tobit model**

The tobit procedure models the association between the independent variable and an underlying latent variable, in this case, the number of reported functional limitations. The longitudinal tobit model can be formulated mathematically as follows:

$$ {{\mathrm{y}}_{\mathrm{i}\mathrm{j}}}^{*}\Big|{\mathrm{b}}_{\mathrm{i}} = \mathrm{x}{'}_{\mathrm{i}\mathrm{j}}\beta + {\mathrm{b}}_{\mathrm{i}} + {\mathrm{e}}_{\mathrm{i}\mathrm{j},}{\mathrm{e}}_{\mathrm{i}\mathrm{j}} \sim \mathrm{N}\left(0,{\sigma}^2\right) $$

$$ {\mathrm{b}}_{\mathrm{i}} \sim \mathrm{N}\left(0,\ \mathrm{D}\right) $$

in which y^*^ is the uncensored latent (i.e. unobservable) dependent variable, β is the parameter, b_i_ is the case-specific random intercept with variance D, i refers to case i, j to the jth measurement within case i.

Tobit regression was estimated with the *xttobit* procedure in Stata. The dependent variable included longitudinal data on disabilities for which the lower limit was set at ‘0’ which corresponds with the reporting of zero disabilities. Since the dependent variable was limited at one side, only a lower limit was needed.

## Abbreviations

ELANE: Elderly and their neighbourhood
IADL: Instrumental activities of daily living
LAPAQ: Physical activity questionnaire of the LASA-study
MET: Metabolic equivalent of task
MVPA: Moderate to vigorous physical activity
